# Magnetic hyperthermia controlled drug release in the GI tract: solving the problem of detection

**DOI:** 10.1038/srep34271

**Published:** 2016-09-27

**Authors:** Joseph C. Bear, P. Stephen Patrick, Alfred Casson, Paul Southern, Fang-Yu Lin, Michael J. Powell, Quentin A. Pankhurst, Tammy Kalber, Mark Lythgoe, Ivan P. Parkin, Andrew G. Mayes

**Affiliations:** 1 Materials Chemistry Centre, Department of Chemistry, University College London, 20 Gordon Street, London, WC1H 0AJ, UK; 2 Centre for Advanced Biomedical Imaging (CABI), Department of Medicine and Institute of Child Health, University College London, London WC1E 6DD, UK; 3 UCL Healthcare Biomagnetics Laboratories, Royal Institution of Great Britain, 21 Albemarle Street, London, W1S 4BS, UK; 4 Institute of Biomedical Engineering, University College London, Gower Street, London WC1E 6BT, UK; 5 School of Chemistry, University of East Anglia, Norwich Research Park, Norwich, Norfolk. NR4 7TJ, United Kingdom

## Abstract

Drug delivery to the gastrointestinal (GI) tract is highly challenging due to the harsh environments any drug- delivery vehicle must experience before it releases it’s drug payload. Effective targeted drug delivery systems often rely on external stimuli to effect release, therefore knowing the exact location of the capsule and when to apply an external stimulus is paramount. We present a drug delivery system for the GI tract based on coating standard gelatin drug capsules with a model eicosane- superparamagnetic iron oxide nanoparticle composite coating, which is activated using magnetic hyperthermia as an on-demand release mechanism to heat and melt the coating. We also show that the capsules can be readily detected via rapid X-ray computed tomography (CT) and magnetic resonance imaging (MRI), vital for progressing such a system towards clinical applications. This also offers the opportunity to image the dispersion of the drug payload post release. These imaging techniques also influenced capsule content and design and the delivered dosage form. The ability to easily change design demonstrates the versatility of this system, a vital advantage for modern, patient-specific medicine.

Specific drug delivery for the gastrointestinal (GI) tract is challenging due to the variety of harsh chemical, physical, enzymatic and mechanical conditions any delivery vehicle has to endure^1^,^2^,^3^,^4^,^5^. This is especially true for orally-administered devices, which must endure peristaltic action of the oesophagus, acidic and mechanical abrasion of the stomach and alkaline, enzymatic and emulsifying conditions in the intestinal tract before payload release. However, oral administration is desirable over intravenous delivery or direct injection into the GI tract due to the easy ingestion and minimally-invasive nature of the process, which benefits patient experience.

Traditional orally-administered drug delivery vehicles rely on changes in internal environment *in vivo* to effect release such as changes in pH or enzymatic conditions^6^,^7^,^8^,^9^ or *via* timed release^10^,^11^,^12^. Often, the use of internal stimuli can lead to unspecific and staggered drug release due to patient-specific differences in GI tract environment, which has proliferated the development of externally triggered systems for modern precision medicine^13^.

The use of an external stimulus to effect drug release is desirable since a physician can control exactly where and when a drug payload is released. This approach has been widely explored, using stimuli such as X-rays^14^, ultrasound^15^,^16^,^17^,^18^, light^19^,^20^,^21^ and by way of magnetic hyperthermia^22^,^23^,^24^,^25^,^26^. For the GI tract, the use of magnetic hyperthermia has significant advantages over other methods of targeted drug release, particularly the use of radiofrequency electromagnetic radiation as the external stimulus which has minimal risk of subsequent complications or harmful side-effects^27^,^28^,^29^,^30^,^31^. The MACH hyperthermia coil used in this study was itself hand held and cooled internally with water in order to prevent any surface tissue damage.

We have found in our previous work that the heat generated from the stimulation of superparamagnetic iron oxide nanoparticles (SPIONs) embedded in an hydrocarbon waxy matrix by radiofrequency was sufficient to melt the matrix in air and water rapidly^22^,^23^. A gelatin drug capsule, loaded with a desired drug payload, was then coated in the nanoparticle-wax composite, forming the drug delivery vehicle (Fig. 1). The observed rapid release on application of an alternating radiofrequency field is highly desirable as the drug payload is released directly to the site of action, without the need of further, molecular or protein-aided targeting.

The disadvantage of using an external stimulus is the need to determine the location of the drug delivery vehicle so that the release stimulus can be provided at the correct time and place to initiate the required release. A suitable detection method would need to be rapid, since if the targeted area is missed, the targeted delivery has failed in that treatment cycle (although in this case it would still have the advantage that the drug would not have been released at all, compared with timed or intrinsic systems where late release would provide a therapeutically-ineffective dose to the patient). It would also be able to determine definitively the position of the vehicle and ideally also confirm if the vehicle had released it’s payload after the stimulus was applied. The premise of our proposed drug delivery system is based on the use of cheap, readily available and pharmaceutically- approved reagents^22^,^23^. Therefore the use of an inexpensive, reliable and easily-applicable detection method would be ideal; especially if it is already used routinely in hospital examinations and would require minimal manipulation of the drug delivery vehicle or payload.

To that end, herein we describe solutions to the problem of detection of the capsule and therefore determination of the release time and location using simulated soft tissue phantoms together with X-ray computed tomography (CT) and magnetic resonance imaging (MRI). MRI provides high resolution, non-invasive anatomical images, as well as sensitive detection of iron oxides – suiting their use together for various biomedical applications^32^,^33^. Due to the high electron density of iron oxides compared to soft tissue, their inclusion in the capsule coating also provides a detection mechanism for CT, which is typically a faster and more cost-efficient imaging technique than MRI.

We then go on to demonstrate how inert, clinically-approved contrast agents such as BaSO_4_ and Iohexol could be included within a capsule payload to enable visualisation of its release. By using CT contrast agents with physical properties that approximate the drug of interest, we suggest that their dispersal and trafficking in the gut could be followed after hyperthermia-mediated release. Finally, we demonstrate the versatility of the above capsule construction and imaging techniques in the production and detection of an alternative drug delivery vehicle design that was constructed without the use of a gelatin capsule template.

## Experimental

### Materials

Barium sulphate (98%), eicosane (99%), octacosane (99%) were purchased from Acros Organics. Oleic acid (technical grade, 90%), calcium carbonate (99%), agar (powder, for microbiology), iron (II, III) oxide (nanopowder, 50–100 nm particle size (SEM), 97% trace metals basis, used as a magnetite XRD standard) and Crystal Violet (hexamethylpararosaniline chloride, >90%) were purchased from Sigma-Aldrich. Iron(III) chloride hexahydrate (98%) was purchased from Alfa Aesar reagents. Iron(II) sulphate heptahydrate (98%), sodium hydroxide pellets (Analytical reagent grade) and ammonium hydroxide solution (28% in water) were purchased from Fisher Scientific, and perfluorooctyl bromide (PFOB, 97%) from Fluorochem Ltd. Iohexol (Omnipaque 300) was obtained from GE healthcare. Aspirin tablets were bought commercially from Boots Ltd. Laboratory solvents were purchased from Sigma Aldrich Limited and were of analytical grade. Aqueous solutions were prepared using UHQ deionised water with a resistivity of not less than 18.2 MΩ cm^−1^. N_2_ (oxygen free) gas was purchased from BOC Ltd. and used as received. Gelatin capsules were purchased from CapsulCN, China.

## Methods

### Synthesis of iron oxide nanoparticles

Iron oxide nanoparticles were synthesised using a co-precipitation method based on the protocol outlined by Lopez-Lopez *et al*.^34^. Briefly, iron(II) sulphate heptahydrate (8.97 g, 32.3 mmol) and iron(III) chloride hexahydrate (17.08 g, 61.3 mmol) were dissolved in 90 and 100 ml degassed deionised water respectively. These solutions were combined under vigorous stirring and the pH increased to 10 using 30% v/v ammonia solution (diluted from conc., 28% in water), resulting in the formation of the Fe_3_O_4_ nanoparticles. Oleic acid (8 ml, 2.53 mmol) was added, and the solution continued to be vigorously stirred for 1 hour. The suspension was then heated to 95 °C at a constant rate of 2 °C min^−1^, and left to cool to room temperature as soon as 95 °C was reached. The pH was then decreased to 5 using sulphuric acid (45%, 8.5 ml) and left to coagulate (*ca.* 30 min). After removal of liquid, the resulting black solid was washed four times with water and then once with acetone, before drying *in vacuo*.

### Capsule coating

Capsules were coated with a molten eicosane/SPION mixture according to our previous work with modifications^22^,^23^. Briefly, the eicosane/SPION layer was applied to the capsules *via* a paint brush using an homogenised molten SPION (at a chosen concentration) in eicosane (*ca.* 50 °C) dispersion. A complete coating was achieved by waiting for the molten layer to cool before applying another coat. The process was repeated for the addition of subsequent layers up to a total of 3 layers (1.5 mm thickness). It was found that a 13:7 (wt:wt) SPION:eicosane ratio was the optimal, as the eicosane/SPION mixture could still be easily handled, melted and used as a coating, yet had a high SPION content.

### Hyperthermia release

Capsule content release was effected by magnetic hyperthermia in air, or whilst suspended in agar or water phantoms (*vide infra*).

### Imaging in agar and water phantom media

Agar phantoms were prepared from 2% w/v agar dissolved by heating to boiling (~100 °C) in distilled water, and set into 50 mL Falcon tubes. Capsules were manually embedded in the agar just before the setting temperature was reached.

Before coating, capsules were loaded with an arbitrary amount (*ca.* 0.1 g) of a contrast agent (barium sulphate, perfluorooctyl bromide or lyophilized Iohexol). Capsules required an extra coating of octacosane (m.p. 57–62 °C) to prevent melting of the coating under the high temperature (40 °C) of the agar phantom before setting. This was not required for samples in water phantoms.

### Instrumentation

Transmission electron microscopy (TEM) images were obtained using a high resolution TEM Jeol 2100 with a LaB_6_ source operating at an acceleration voltage of 200 kV. Images were recorded on a Gatan Orius Charge-coupled device (CCD). Samples were drop-cast onto carbon-coated 400 mesh copper grids (Agar Scientific Limited, UK). Dynamic Light Scattering (DLS) was measured using a Malvern Zetasizer Nano-ZS. Powder X-ray diffraction (pXRD) patterns were recorded using a Stoe (Mo) StadiP diffractometer with a Mo X-ray source (Mo tube 50 kV 30 mA, λ = 0.70926 Å), monochromated (Pre-sample Ge (111) monochromator selects Kα1 only) and a Dectris Mython 1 k silicon strip detector covering 18° 2θ. Magnetisation data was taken using a Quantum Design MPMS Super conducting Quantum Interference Device (SQUID) VSM Magnetometer (San Diego, USA) at 300 K using a field range of ± 7 T. Hyperthermia experiments were undertaken using a MACH system (Magnetic Alternating Current Hyperthermia) designed and built by Resonant Circuits Limited (3.52 kA/m and the frequency is 990 kHz)^35^. The temperature was monitored using a fluoroptic [fibreoptic] temperature probe (Luxtron FOT Lab Kit, Lumasense California USA). Thermal images were recorded with an Infratec (Germany) VarioCAM HR research 780 with 30 mK thermal resolution and 1280 × 960 spatial resolution. Capsules were mounted in a weighing boat in air, or loaded with sand (to increase their density) for submerged experiments. X-Ray photoelectron spectroscopy (XPS) was performed using a Thermo Scientific K-alpha spectrometer with monochromated Al Kα radiation, a dual beam charge compensation system and constant pass energy of 50 eV (spot size 400 μm). Survey scans were collected in the range 0–1200 eV. High-resolution peaks were used for the principal peaks of Fe (2p), O (1 s) and C (1 s). Magnetic Resonance Images were obtained using a 1T Brüker ICON desktop MRI system (Brüker BioSciences Corporation, Ettlingen, Germany), interfaced to an operating console running Paravision 5 software (Bruker). A 44 mm rat body solenoid RF coil (Brüker) was operated on transmit/receive mode. Multi-slice images were acquired using a spoiled gradient echo sequence with TE of 6.2 ms and a TR of 387 ms, 2 averages, and an 80° flip angle. Images were acquired for a 4 × 4 cm field of view with 256 × 256 in plane resolution, and 20 slices of 2 mm thickness with 2.25 mm separation. Total scan time was 3 minutes 19 seconds per sample. Volume rendering was done using ImageJ software. CT images were obtained using a Nanoscan PET/CT (Mediso), with tube voltage of 50 keV, 30 ms exposure, and 720 projections, operated using Nucline 2 software (Mediso). Total scan time was 3 minutes 46 seconds per sample. Reconstructions were done with Nucline 2 software, analysis and volume rendering was done in Vivoquant (inviCRO) and Interview Fusion (Bartec) software.

## Results and Discussion

Iron oxide nanoparticles were synthesised using a co-precipitation method, by the precipitation of magnetite Fe_3_O_4_ from an aqueous solution of ferrous and ferric salts in basic conditions. The precipitate was subsequently treated with oleic acid to ensure the particles were readily dispersible in organic media. This ensured even particle distribution throughout the wax coating matrix and even, rapid heating throughout. This is important as it minimises patient exposure to the hyperthermia magnetic field, and decreases overall treatment time.

**Figure 1:**
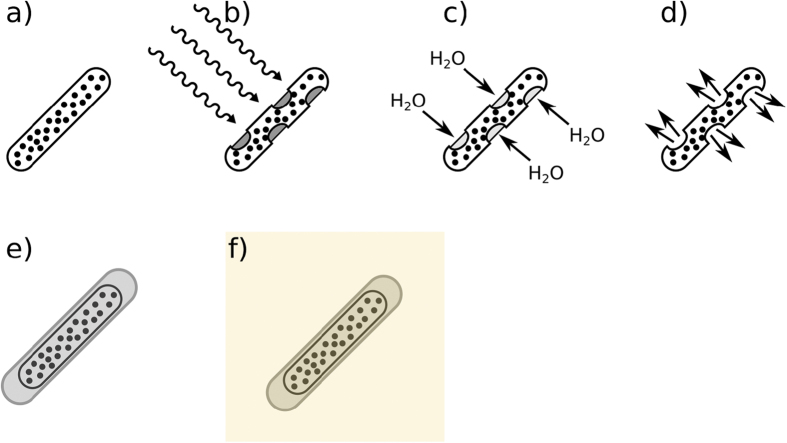
Process of capsule synthesis and mechanism of drug release. (**a**) demonstrates a SPION-wax coated drug capsule which, when at the desired point of release, is then irradiated with radiofrequency, heating the SPIONs and melting the wax coating, (**b**). Water ingress then dissolves the gelatin drug capsule template, (**c**) before release of the drug payload, (**d**). In order to prevent wax melting during incorporation into an agar phantom for MRI/CT imaging, a thin layer of pure octacosane (m.p. 57–62 °C) was added *via* dip-coating, (**e**). The octacosane-SPION-wax coated capsule was then incorporated into an agar phantom at ~40 °C, (**f**).

The co-precipitation method for the synthesis of iron oxide nanoparticles is a scalable method for the rapid production of magnetic material. However, products are typically highly polydisperse (Fig. 1a)) when compared to hydrophobic ligated magnetic nanoparticles synthesised by thermal decomposition methods^36^,^37^,^38^,^39^,^40^,^41^. The inherent polydispersity however can be seen as an advantage due to the array of commercially-available and bespoke magnetic hyperthermia apparatus, each generating different field strengths and frequencies^42^. Therefore, a polydisperse SPION sample is more likely to be compatible with a wider range of hyperthermia instruments.

**Figure 2:**
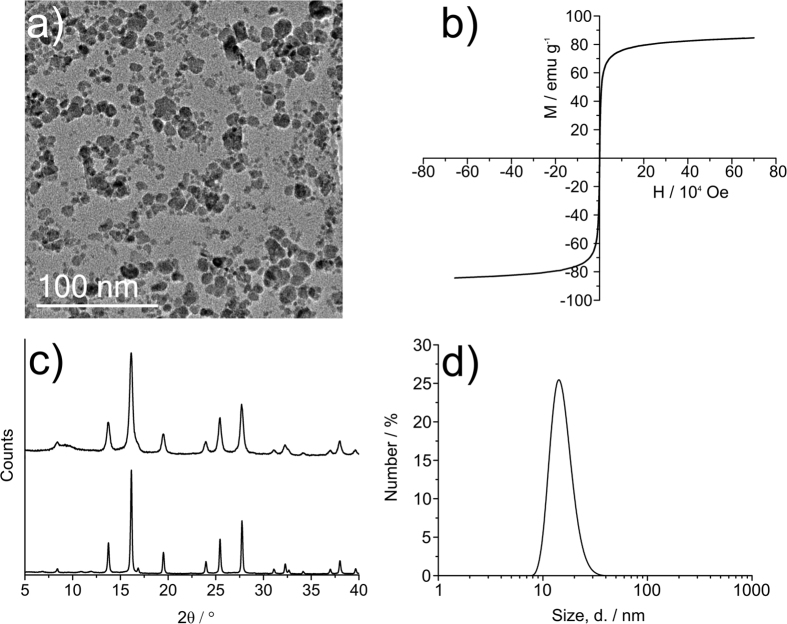
(**a**) TEM micrograph of 8.5 nm iron oxide nanoparticles showing polydispersity, with an average core size of about 8.5 nm, (**b**) SQUID magnetometry measurement showing a saturation magnetisation of 84.7 emu g^−1^ at 300 K and the absence of hysteretic behaviour indicating superparamagnetism, (**c**) an XRD pattern of iron oxide nanoparticles (compared with a magnetite standard below) and (**d**) an average DLS size (by number, 15.3 ± 3.61 nm).

TEM analysis of the nanoparticles obtained by co-precipitation demonstrates a polydisperse sample with an average size of 8.5 nm and a standard deviation of 3.6 nm. This is supported by the solvodynamic radius of 15.3 ± 3.61 nm obtained by DLS (Fig. 2d), after washing and re-dispersion in *n*-hexane. This is a larger value than that obtained for the core size by TEM as this includes the oleic acid ligand corona. High resolution TEM analysis (Figure S2) gave lattice *d*-spacings of 0.90 nm and 0.248 nm, which are assigned to the <220> and <311> planes of magnetite.

The XRD pattern of the nanoparticles was assigned as iron oxide, presumed to be that of magnetite (Fig. 2c), ICSD 26410, magnetite Fe_3_O_4_^43^. Note that an Fe_3_O_4_ control (97%, Sigma Aldrich) was run as a control and that the radiation used was Molybdenum Kα (λ = 0.70926 Å).

The magnetic properties of the nanocrystals were analysed by SQUID magnetometry. Saturation magnetisation values of 84.7 emu g^−1^ and 21.7 emu g^−1^ were recorded for 21.5 mg of SPION sample and 13.1 mg of the sample dispersed in eicosane wax (13:7 wt:wt) respectively. The complete absence of magnetic hysteresis and zero coercivity in any of the samples and their small size as evidenced by TEM and DLS, indicated that the nanoparticles are superparamagnetic at 300 K. XPS analysis of the SPIONs showed the presence of iron and oxygen (Fe 2p_3/2_, binding energy 710.9 eV) and (O1 s, binding energy 530.1 eV) assigned as Fe_3_O_4_^44^,^45^,^46^,^47^. Further O1 s peaks are assigned as FeOOH (binding energy 531.8 eV) and O in organic species (presumed to be oleic acid, COO, binding energy 533.4 eV)^48^.

As-synthesised SPIONs were subsequently dispersed in molten eicosane at 50 °C before application of the coating *via* a paintbrush to the capsule surface. Around 3 complete coatings were necessary to ensure the capsule was impervious to water. Capsules were heated in both water and air to demonstrate effective melting of the capsule coating. In our previous work, the authors investigated the composition, melting properties and stability under physiological conditions of various wax-SPION composite coatings^22^,^23^. We concluded that a wax containing a mixture of eicosane and docosane (40/60 w/w, m.p. 42 °C) would be the best candidate in order to melt above body temperature, but not be too high as to damage extraneous tissue. In all wax composites trialled, the SPIONs exhibited good dispersion in all wax composites, therefore making the melting point of the coating tuneable according to the melting point of the wax used. In this study, we utilised eicosane simply as a model hydrocarbon wax for imaging purposes, as *in vivo* tests would require the aforementioned higher melting point eicosane-docosane composite.

The materials used to synthesise the capsules and capsule coatings are pharmaceutically approved (*i.e.* all the chemicals used are listed in the British Pharmacopoeia either as medications in their own right (e.g. ferrous sulphate B.P. for treatment of anaemia), as components of other medications (e.g. oleic acid in emulsions and ointments) or in less refined forms (e.g. eicosane is a component of white soft paraffin/petroleum jelly)).

**Figure 3:**
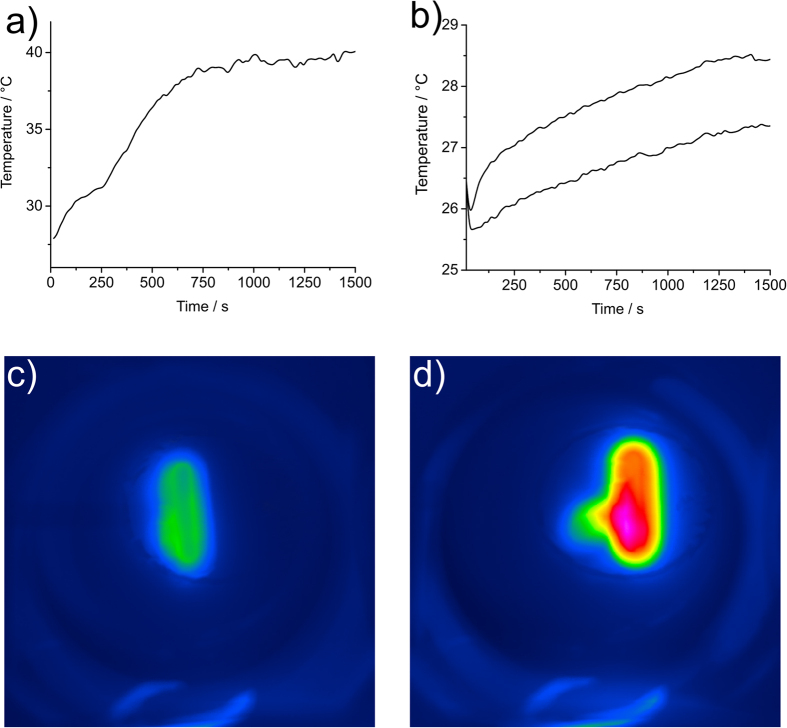
(**a**) Graph of heating an eicosane- SPION coated capsule in air demonstrating heating behaviour commensurate with the latent heat of melting of eicosane and (**b**) submerged in water. Melting was difficult to observe *via* an attached thermocouple as the thermocouple detached as the material melted, (**c**,**d**) are thermal images showing progression of melting with time, at 105 seconds in (**c**) and 285 seconds in (**d**) for the capsule heated in air.

Temperature versus time data extracted from the thermal imaging camera was used for the capsule in air (Fig. 3a). Although an attached fibreoptic thermocouple was used for the heating of the capsule in water in (Fig. 3b), the disintegrating nature of the melting coating made this exacting, so where appropriate, the thermal camera was used. As seen previously^22^,^23^, the heating curve in air displayed initial heating to a plateau. This plateau is due to the energy required to supply the latent heat of melting, hence a plateau was observed as the material undergoes phase transition to a liquid. The incorporation of SPIONs tend to lower the melting temperature of the composite from the literature melting point of eicosane (35–37 °C), although incorporation of higher molecular mass alkanes again raises the melting point^23^. Rapid heating was observed as the solid eicosane matrix became liquid.

In (Fig. 3b), heating and melting of the capsule in water was achieved even though the fibreoptic thermocouple attached to the coating decoupled from the disintegrating coating and was subsequently cooled by the surrounding water (see photographic stills S4.3–4.5).

The ability of a 1 Tesla MRI system to detect the iron oxide-wax-coated capsules was assessed. The presence of iron oxides in tissue should produce a hypo-intense signal due to the dephasing of surrounding water proton spins, proving a potential clinically-applicable means to detect the capsules and their transit in patients. To approximate the surrounding soft tissue contrast in the gut, capsules were embedded in a 50 ml volume of 2% agar prior to imaging.

**Figure 4:**
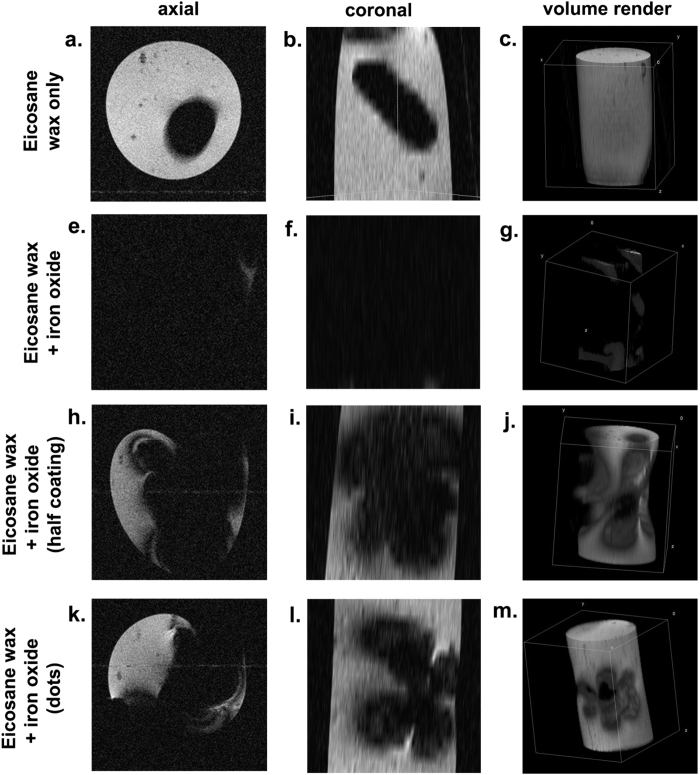
Magnetic resonance images showing signal hypointensity produced by iron oxide wax-coated capsules compared to capsules coated in the wax only. Axial (**a**,**e,h,k**), and coronal slices (**b,f,I,l**) through the capsules are shown, as well as a volume rendering of the agar phantom containing the capsules.

Multi-slice images were taken across the capsule and agar phantom, and reconstructed into 3D volumes, Fig. 4. In the capsules coated in wax only, the cavity and coating of the capsule can be seen as a hypo-intense region against the background signal provided by the agar (Fig. 4a_c). In the capsules coated in the wax-iron oxide composite, however, the hypo-intense region was enlarged, resulting in a drop out of signal from all but the top edges of the agar phantom (4d to f). Coatings on only one half of the capsule, or as dots on the capsule, produced intermediate volumes of hypo-intensity between those of the capsules with wax only, and a full coating of iron oxide-wax (4 h to m). The large amount of iron in the coatings required for hyperthermia-mediated drug release precluded the possibility of obtaining accurate T_2_ maps, due to the extreme T_2_ shortening.

**Figure 5:**
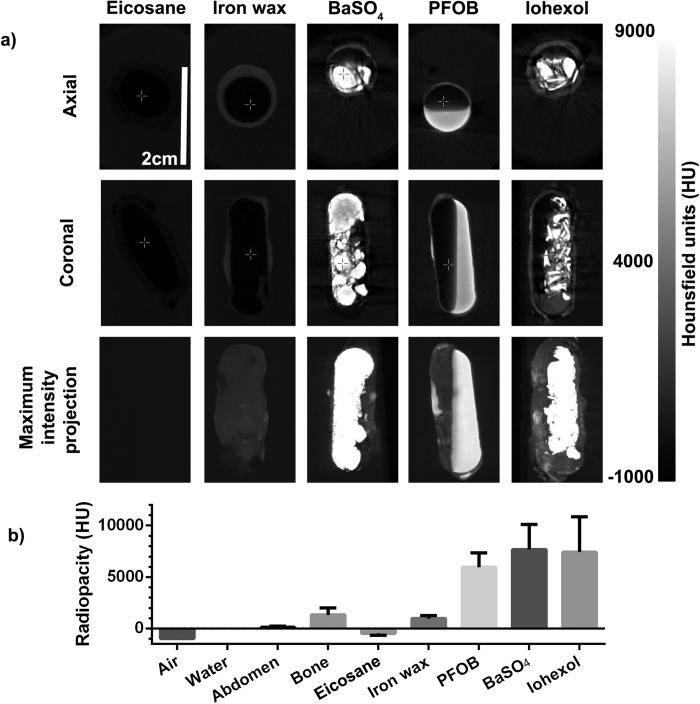
(**a**) CT images showing capsules coated in wax only (Eicosane), iron-oxide wax coating, and the iron oxide-wax coated capsules filled with BaSO_4_, Perfluorooctylbromide (PFOB), and Iohexol. (**b**) Quantification of radiopacity of the capsules shown in (**a**). Bars show mean X-ray absorbance of all pixels contained within the capsule coating or filling, error bars show SD of the whole segmented region in the capsule cavity. The absorbance of air, water, abdomen soft tissue, and bone are provided for reference.

One potential limitation of iron-based contrast agents for MRI is the “blooming” effect, whereby signal hypo-intensity is produced over a greater area than that which contains the contrast agent, Fig. 5. Though this can increase the sensitivity with which iron oxides can be detected, it can also reduce the accuracy of locating the contrast agent, as well as obscuring anatomical features that might be important for reference when targeting delivery. A second limitation is the potential inability to distinguish between the presence of T_2_ contrast agents such as iron oxides, and endogenous areas of signal hypo-intensity and susceptibility artefacts caused by gas/tissue interfaces in the bowel. For these two reasons, we next chose to investigate the incorporation of X-ray CT contrast agents into the capsules, which should provide the advantages of both positive contrast and a less ambiguous mode of detection. Furthermore, the use of CT is significantly cheaper and quicker than MRI in the clinic, providing a better option for translation. CT scanning is sufficiently fast as to give a pinpoint location of the capsule. Gastrointestinal transit time may be increased or decreased with the use of appropriate binding medication or laxatives respectively to aid detection^49^,^50^,^51^,^52^. In this way, hyperosmotic agents increase the water content of the intestine and so can change the environment in which the delivery occurs. This is advantageous in both detection and drug release and diffusion.

We then showed that CT imaging could detect the iron oxide wax coating, which provided signal above that of the simulated soft tissue contrast of the agar phantom, Fig. 5. The X-ray absorbance of the iron-oxide wax was measured at 952 ± 297 HU, which is above the typical soft tissue absorbance of around 30 ± 5 HU^53^, and close to the strong radiopacity of bone (1000 to 1600 HU). In comparison, the eicosane wax without iron oxide gave much lower X-ray absorbance than both the iron oxide wax and soft tissue (−473.798 ± 183.650 HU), showing that it is the addition of the iron oxide to the wax that is responsible for its ability to produce CT contrast^54^. This suggests that X-ray imaging might provide an effective means to track the location and break-up of the iron-oxide wax coated capsule without the addition of any further encapsulated contrast agents. However, this would not provide information on the release and subsequent diffusion of encapsulated drugs.

To demonstrate the ability to image the triggered release of the capsule contents, three different CT contrast agents were encapsulated (Barium sulfate (BaSO_4_), perfluorooctyl bromide (PFOB), and Iohexol), and the capsules imaged with CT, Fig. 5. Of these, BaSO_4_ and Iohexol are in routine clinical use^55^, and the PFOB-based CT contrast agent Imagent BP ^®^ has also been approved for clinical use by the FDA^56^,^57^. These agents therefore provide a safe method for tracking the release of encapsulated contents upon hyperthermia-mediated dissolution of the capsule. CT images revealed that further contrast was provided by the inclusion of these encapsulated CT contrast agents: BaSO_4_ (7672.88 ± 2414.30 HU), PFOB (5950.51 ± 1382.75 HU), and Iohexol (7417.74 ± 3437.47 HU). The increase in radiopacity provided by these was much greater than that of the iron-oxide wax, and several fold that of bone.

**Figure 6:**
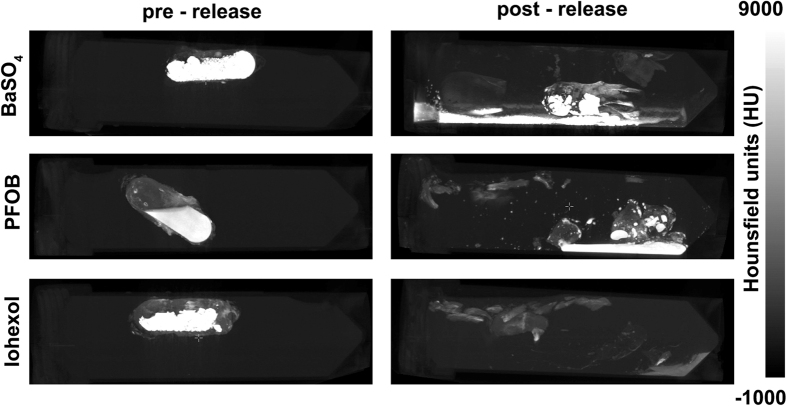
CT images show iron-oxide wax coated capsules containing CT contrast agents before and after triggered release (60 minutes). Post-release images show the distribution of the released contrast agents.

Next, water-based phantoms containing the capsules with CT contrast agents were prepared. For the imaging to provide representative information on drug release, it is important to have contrast agents that behave in a similar way to the encapsulated drug of interest, so that their release from the capsule and absorption or trafficking in the bowel would be similar to that of the drug. For this reason the three CT contrast agents were selected to display different physical behaviour- BaSO_4_ (insoluble, granular), PFOB (liquid, immiscible in water), and Iohexol (powdered, soluble in water). In particular, the PFOB and BaSO_4_ mimic poorly soluble drugs with limited bioavailability, and any dissolution steps any such drug might have to overcome, for example if it was encased in an amphiphilic polymer^58^,^59^,^60^. These were imaged before and after release, to assess the ability of CT to visualise dispersal of the capsule contents. Figure 6 shows that contents release can be visualised with BaSO_4_, PFOB, and Iohexol filled capsules. In the case of BaSO_4_ and PFOB, in which the contents are not soluble in water, the released contents can be seen lining the base of the tube of water into which they have been released. In the case of Iohexol, which is water soluble, it can be seen that the contrast agent has dissolved, confirming that the contents of the capsule have been exposed to the exterior aqueous environment after heating.

**Figure 7:**
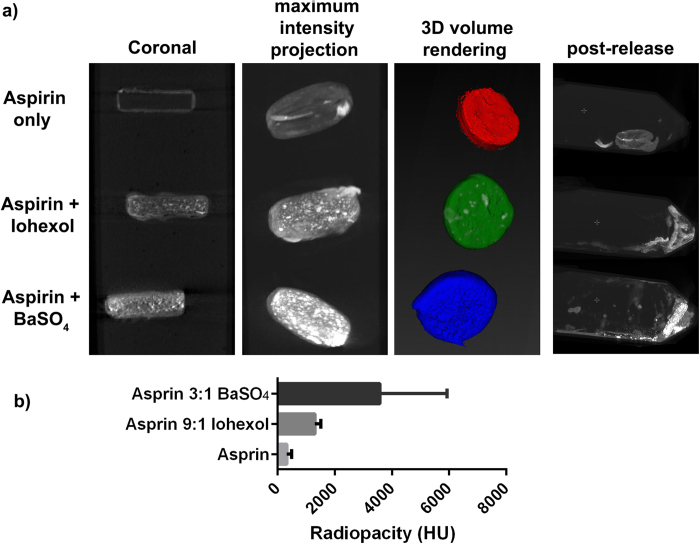
(**a**) CT images showing coronal CT slices, a reconstructed maximum intensity projection, and 3D volume rendering of the Aspirin tablets coated with the iron wax composite, embedded in 2% agar. Addition of Iohexol or BaSO_4_ improved CT visibility. Post-release images showed the degradation of the coating completely and the partial dissolution of the aspirin template. (**b**) Quantification of radiopacity of aspirin tablet with iron wax coating, and aspirin table incorporating 25% w/w BaSO_4_ or 10% w/w Iohexol. Error bars represent the standard deviation in Hounsfield units of all pixels in the 3D region of interest shown segmented in the 3D volume render image in (**a**).

The preceding capsule designs used in Figs 3^_^6 have relied on a gelatin capsule template that is filled with the drug of interest and contrast agent, prior to coating with the waterproof iron-wax composite. However, to simplify the design of the system, a gelatin capsule-free approach was investigated, in which the drug and contrast agent were compacted into a solid tablet, then coated directly in the iron-wax composite. Using this approach, we successfully incorporated BaSO_4_ and Iohexol into a commercial aspirin formulation at 25 and 10% w/w respectively. CT images showed that these tablets also appeared bright on CT images (Fig. 7), again with much higher radiopacity than bone.

Post-release, it was observed that the capsule wax coating was entirely removed from the aspirin template on exposure to an external magnetic field, once again showing the viability of the on-demand, rapid triggered release mechanism. However, due to the largely insoluble nature of the formulation including substances such as calcium carbonate and talc, which is a lubricating agent in the tablet manufacturing process, the aspirin formulation also proved to only be very sparingly soluble in acidic conditions (~pH 3), with the coating protecting the payload (48 hours) without incident until release was effected by hyperthermia. Nevertheless, the incorporation of Iohexol and BaSO_4_ was highly effective in aiding CT imaging in this exemplar system, and with a near limitless number of pharmaceutical formulations possible, the iron oxide wax composite coating has a bright future.

Furthermore, this demonstrates the influence of detection on capsule design, and indeed the versatility of hyperthermia-activated drug delivery coatings, especially in challenging target organs such as the GI tract. In this way, the release of powdered, poorly soluble drugs specifically to locations in the GI tract, locally increasing bio-availability can be realised, with a focus on affordable patient-specific dosage-form design.

## Conclusion

Hyperthermia-controlled drug release has been demonstrated using an eicosane/SPION composite coated delivery vehicle. Our robust iron oxide wax capsule coating can provide contrast on both X-ray computed tomography (CT), and magnetic resonance imaging, providing a clinically- applicable means to monitor transit of the delivery vehicle in order to control release in specific portions of the GI tract. We have also demonstrated the feasibility of MRI for detection, however superior cost effectiveness and throughput (seconds to minutes per scan, depending on the application)^61^ make CT imaging more desirable. By encapsulating CT contrast agents into the vehicle design, it is possible to image contents release using CT to confirm drug delivery at the target site. It is noteworthy that the eicosane/SPION composite itself exhibited CT contrast comparable to that of bone (952 ± 297 HU vs. 1000 to 1600 HU for strong radiopacity of bone) without the addition of contrast agents such as BaSO_4_ and Iohexol.

Detection has also influenced capsule design, with iron oxide wax applied so as to create a unique shape for MRI, thus readily distinguishing it from air pockets in the gut. Other pharmaceutical formulations, such as the use of powdered drugs with contrast agents pressed into a pellet were also examined. This enables the rapid increase in local bio-availability of poorly soluble drugs, with a focus on affordable patient-specific capsule design. These improvements, along with the intrinsic simplicity of the vehicle and release mechanism and detection strategies demonstrate the versatility of our system and will aid future translation to the clinic. The approach outlined in this work is generic, so in principle almost any pharmaceutical formulation could be packaged and released in this way. It would also be possible to engineer much more sophisticated multi-compartment or multi-component devices with additional features to enhance drug release or dissolution or minimise the loading of iron-oxide wax coating to reduce the required magnetic field exposure, though it should be emphasised that the fields used in this work are well within acceptable limits for clinical application.

## Additional Information

**How to cite this article**: Bear, J. C. *et al*. Magnetic hyperthermia controlled drug release in the GI tract: solving the problem of detection. *Sci. Rep.* **6**, 34271; doi: 10.1038/srep34271 (2016).

## Supplementary Material

Supplementary Information
