# Supplementary material for: Magnetic hyperthermia controlled drug release in the GI tract: solving the problem of detection
Source: Sci Rep. 2016 Sep 27;6:34271. doi: 10.1038/srep34271 (PMC5037467; doi:10.1038/srep34271)
Supplement: Supplementary Information [file srep34271-s1.pdf]

“Magnetic hyperthermia controlled drug release in the GI tract: solving the problem of detection”- Supporting Information

Joseph C. Bear,*[1] P. Stephen Patrick,[2] Alfred Casson,[1] Paul Southern,[3] Fang-Yu Lin,[3] Michael J. Powell,[1] Quentin A. Pankhurst,[3,4] Tammy Kalber,[2] Mark Lythgoe,[2] Ivan P. Parkin[1] and Andrew G. Mayes*[5]

[1] Materials Chemistry Centre, Department of Chemistry, University College London, 20 Gordon Street, London, WC1H 0AJ, UK.

[2] Centre for Advanced Biomedical Imaging (CABI), Department of Medicine and Institute of Child Health, University College London, London WC1E 6DD, UK.

[3] UCL Healthcare Biomagnetics Laboratories, Royal Institution of Great Britain, 21 Albemarle Street, London, W1S 4BS, UK.

[4] Institute of Biomedical Engineering, University College London, Gower Street, London WC1E 6BT, UK.

[5] School of Chemistry, University of East Anglia, Norwich Research Park, Norwich, Norfolk. NR4 7TJ, United Kingdom.

Email: joseph.bear.11@ucl.ac.uk ; andrew.mayes@uea.ac.uk

1. XPS spectra

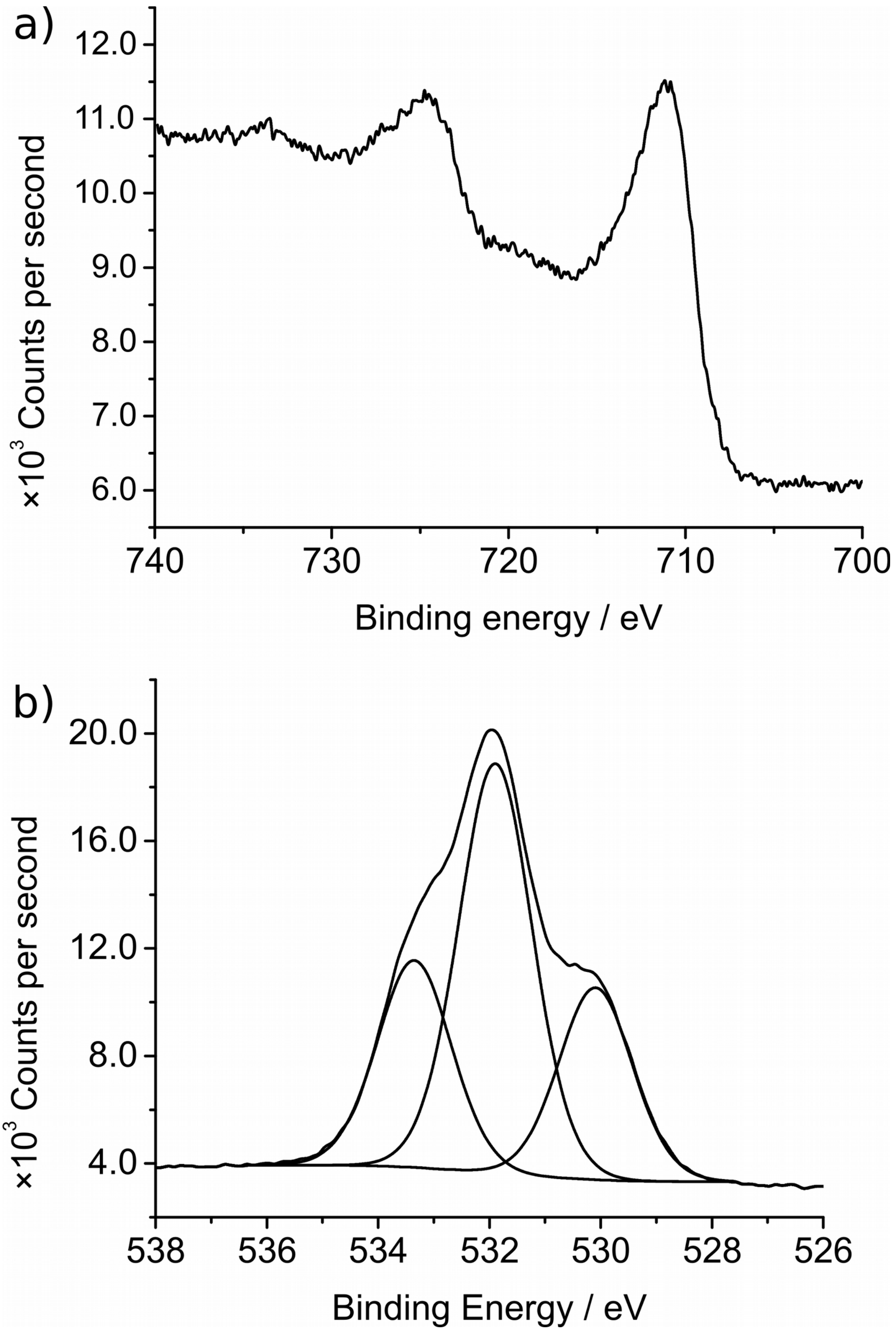

Figure S1: High resolution XPS spectra of SPIONs formed by co-precipitation. a) Fe 2p region and b) O1s showing the fitted environments of 530.1 eV assigned as Fe_3O_4, 531.8 eV assigned as FeOOH and 533.4 eV assigned as oleic acid, COO, 533.4 eV.

2. HRTEM images

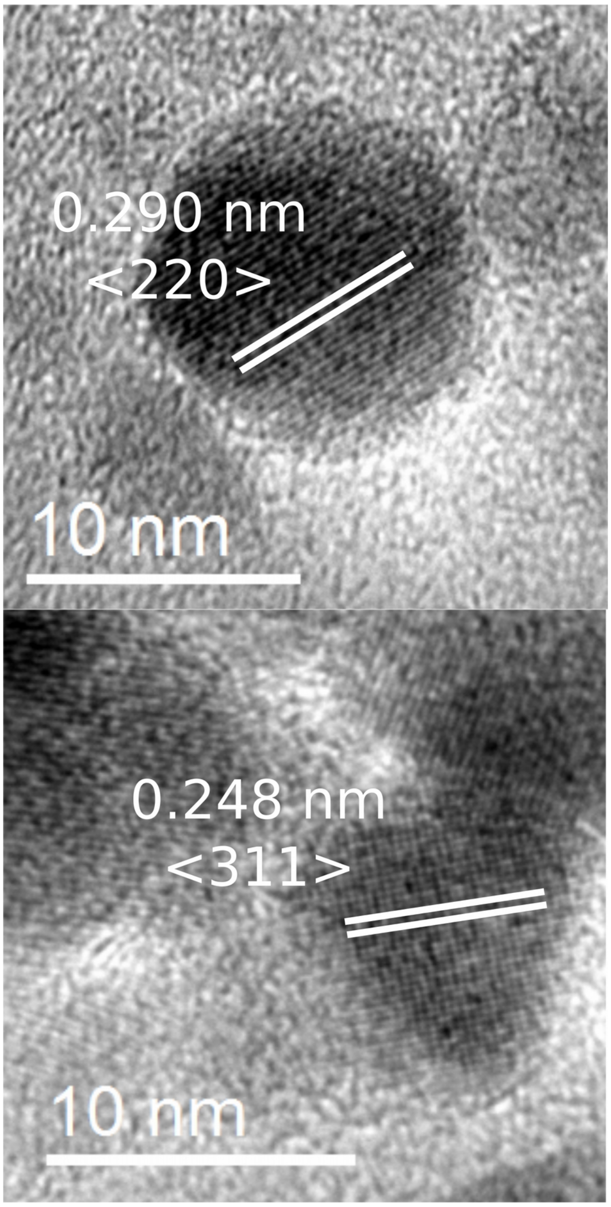

Figure S2: High resolution transmission electron microscope images of as-synthesised iron oxide nanoparticles. Lattice *d*-spacings of 0.290 nm and 0.248 nm are assigned to the <220> and <311> planes of magnetite Fe_3O_4.

3. Additional XRD patterns

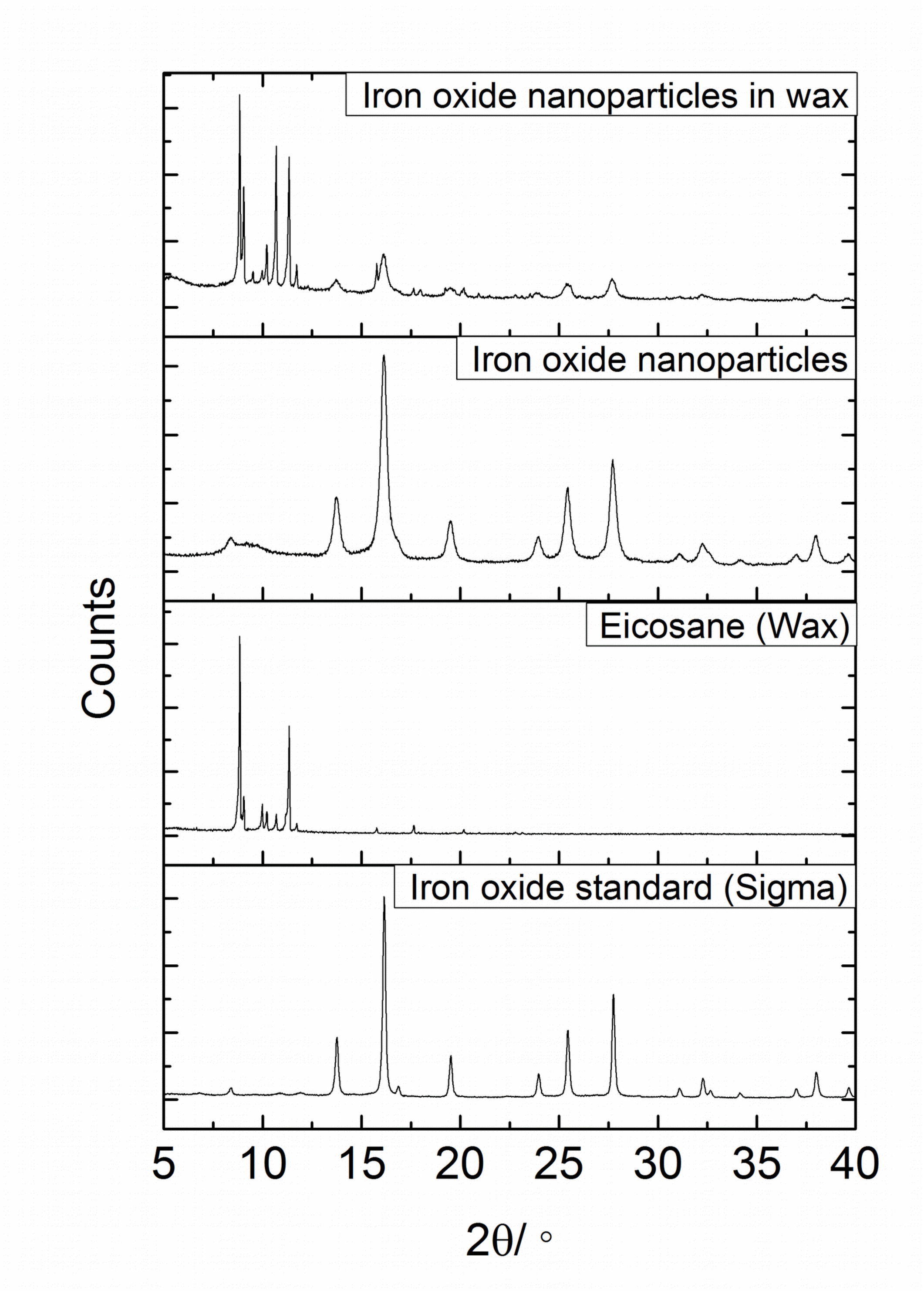

Figure S3: XRD patterns of: (from bottom) Magnetite Fe_3O_4 standard (Sigma Aldrich, nanopowder, 50-100 nm particle size (SEM), 97% trace metals basis), eicosane (Sigma Aldrich, 99 %), SPIONs synthesised using co-precipitation and SPION / eicosane wax composite.

4. Photographs

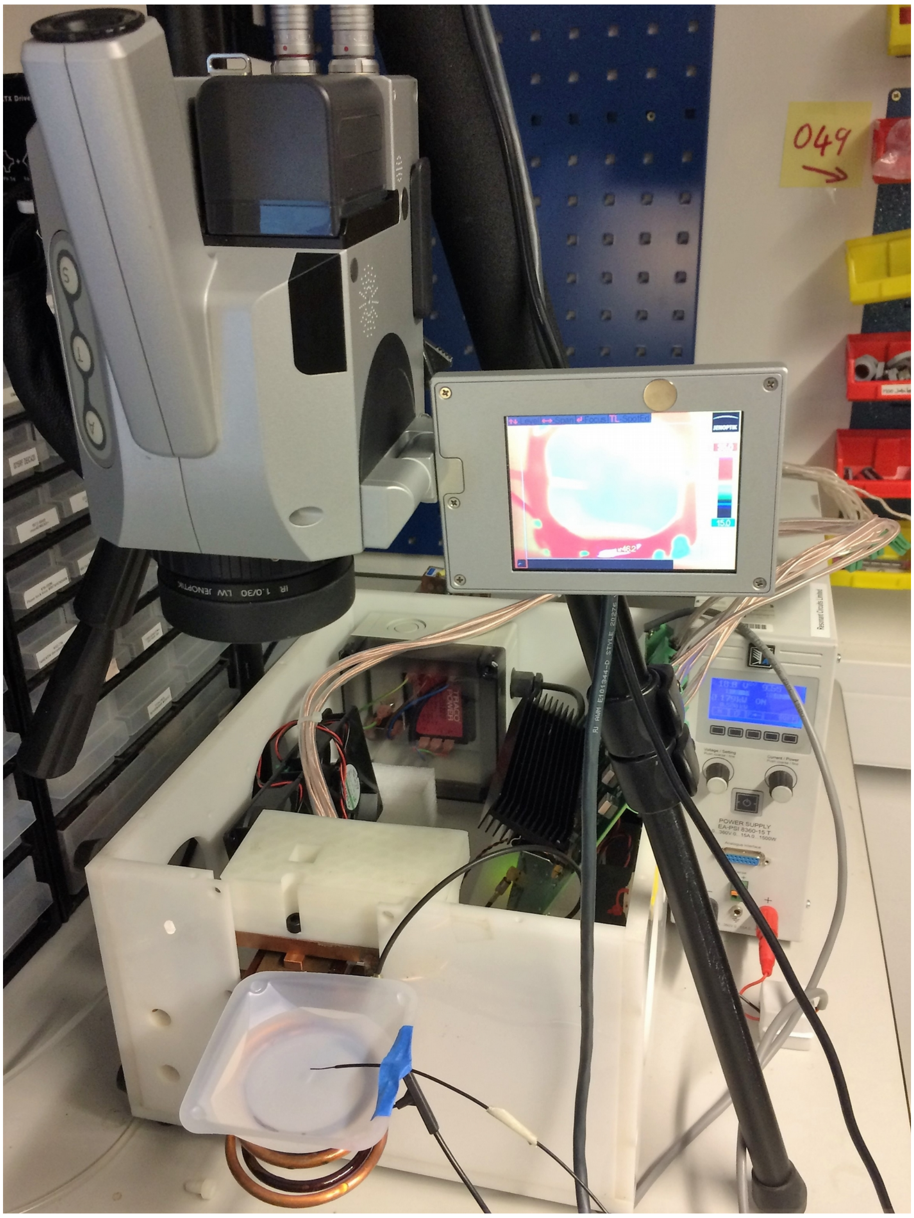

Figure S4.1: The MACH hyperthermia system set-up with the VarioCAM HR research 780 thermal imaging camera mount.

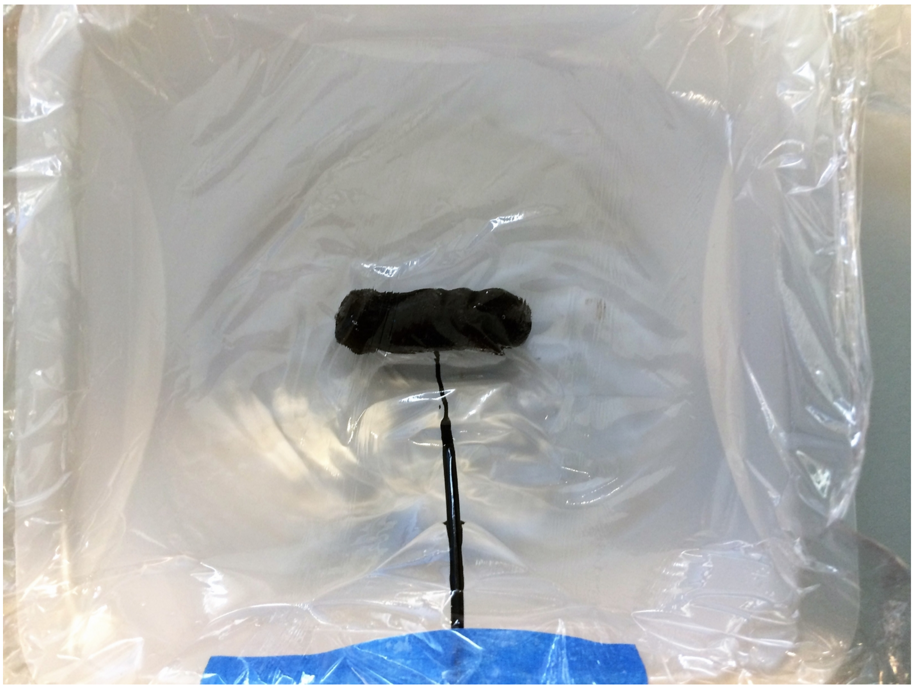

Figure S4.2: Submerged eicosane-SPION coated capsule with fibre-optic probe attached.

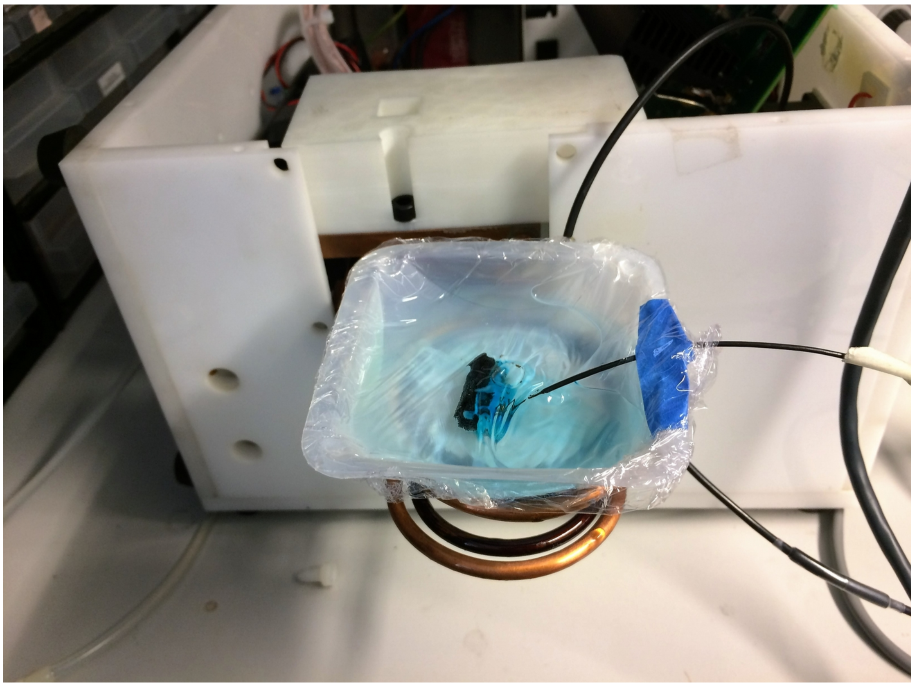

Figure S4.3: Submerged eicosane-SPION coated capsule with fibre-optic probe attached, post release.

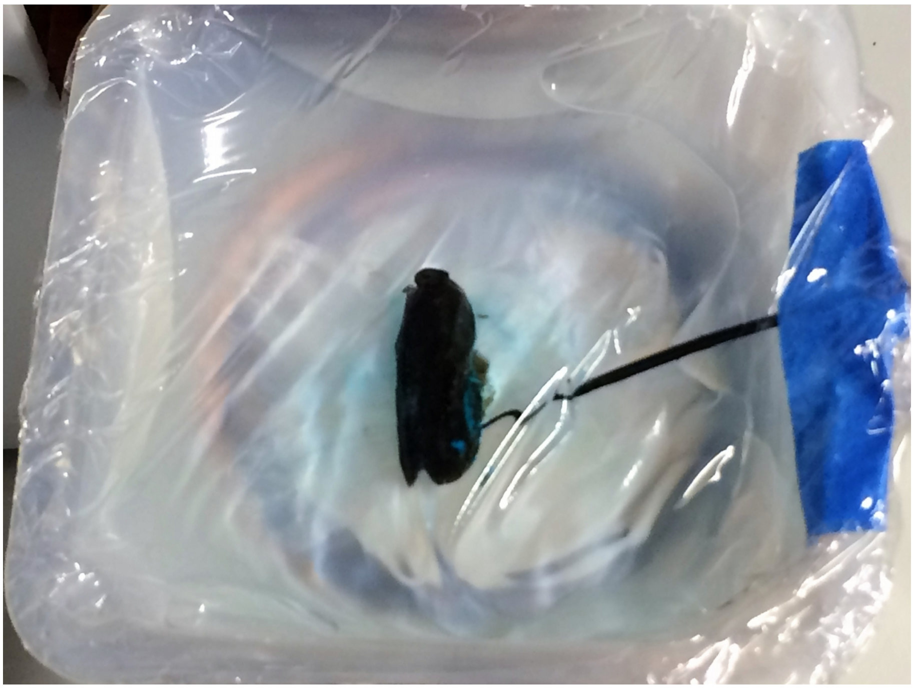

Figure S4.4: A submerged eicosane-SPION coated capsule with fibre-optic probe coming unattached during release.

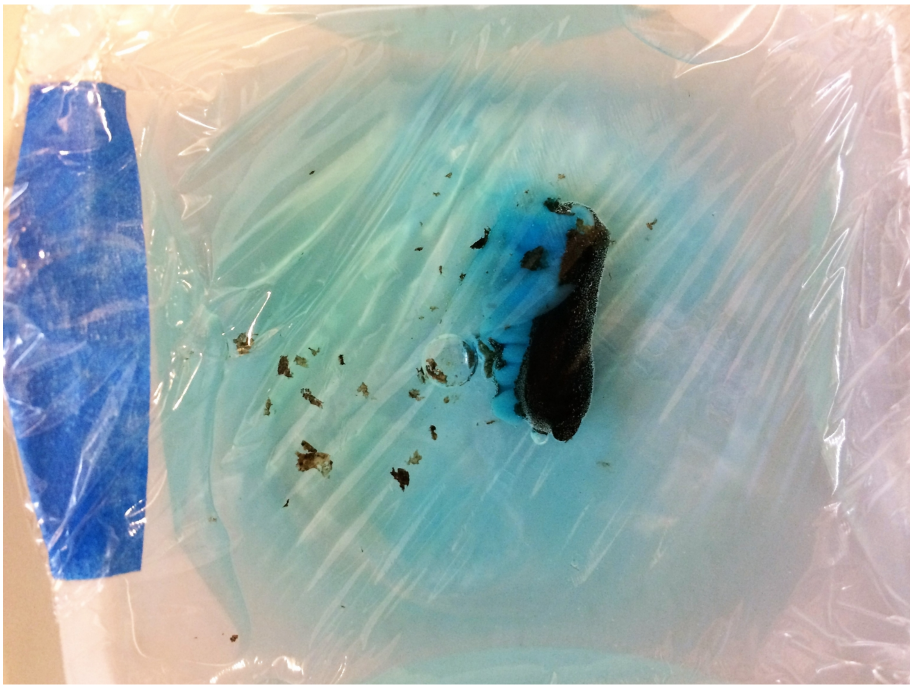

Figure S4.5: A submerged eicosane-SPION coated capsule with fibre-optic probe unattached, post activation.
